# FT-IR-cPAS—New Photoacoustic Measurement Technique for Analysis of Hot Gases: A Case Study on VOCs

**DOI:** 10.3390/s110505270

**Published:** 2011-05-16

**Authors:** Christian Bernd Hirschmann, Niina Susanna Koivikko, Jussi Raittila, Jussi Tenhunen, Satu Ojala, Katariina Rahkamaa-Tolonen, Ralf Marbach, Sarah Hirschmann, Riitta Liisa Keiski

**Affiliations:** 1 Photonic Devices and Measurement Solutions, VTT Technical Research Centre of Finland, Kaitoväylä 1, FI-90570 Oulu, Finland; E-Mails: jussi.tenhunen@vtt.fi (J.T.); satu.ojala@oulu.fi (S.O.); katariina.rahkamaa-tolonen@vtt.fi (K.R.-T.); ralf.marbach@vtt.fi (R.M.); 2 Mass and Heat Transfer Process Laboratory, Department of Process and Environmental Engineering, University of Oulu, FI-90014 Oulu, Finland; E-Mails: niina.koivikko@oulu.fi (N.S.K.); sarah.hirschmann@oulu.fi (S.H.); riitta.keiski@oulu.fi (R.L.K.); 3 Gasera Ltd., Tykistökatu 4, FI-20520 Turku, Finland; E-Mail: jussi.raittila@gasera.fi (J.R.)

**Keywords:** volatile organic compound (VOC), photoacoustic spectroscopy (PAS), science based calibration (SBC), elevated temperature measurement

## Abstract

This article describes a new photoacoustic FT-IR system capable of operating at elevated temperatures. The key hardware component is an optical-readout cantilever microphone that can work up to 200 °C. All parts in contact with the sample gas were put into a heated oven, incl. the photoacoustic cell. The sensitivity of the built photoacoustic system was tested by measuring 18 different VOCs. At 100 ppm gas concentration, the univariate signal to noise ratios (1σ, measurement time 25.5 min, at highest peak, optical resolution 8 cm^−1^) of the spectra varied from minimally 19 for o-xylene up to 329 for butyl acetate. The sensitivity can be improved by multivariate analyses over broad wavelength ranges, which effectively co-adds the univariate sensitivities achievable at individual wavelengths. The multivariate limit of detection (3σ, 8.5 min, full useful wavelength range), *i.e.*, the best possible inverse analytical sensitivity achievable at optimum calibration, was calculated using the SBC method and varied from 2.60 ppm for dichloromethane to 0.33 ppm for butyl acetate. Depending on the shape of the spectra, which often only contain a few sharp peaks, the multivariate analysis improved the analytical sensitivity by 2.2 to 9.2 times compared to the univariate case. Selectivity and multi component ability were tested by a SBC calibration including 5 VOCs and water. The average cross selectivities turned out to be less than 2% and the resulting inverse analytical sensitivities of the 5 interfering VOCs was increased by maximum factor of 2.2 compared to the single component sensitivities. Water subtraction using SBC gave the true analyte concentration with a variation coefficient of 3%, although the sample spectra (methyl ethyl ketone, 200 ppm) contained water from 1,400 to 100k ppm and for subtraction only one water spectra (10k ppm) was used. The developed device shows significant improvement to the current state-of-the-art measurement methods used in industrial VOC measurements.

## 1. Introduction

In environmental pollutant and exhaust gas analyses, the emitted gas concentrations can be very low, and thus difficult to qualify and even more challenging to quantify. In spite of the technical progress of recent years, one of the most demanding and still unresolved needs is the reliable measurement of volatile organic compounds (VOC) [1,2]. VOC emissions cause atmospheric pollution and damage the stratospheric ozone layer. By reacting with nitrogen oxides, they create smog in the lower atmosphere which reduces the quality of air and finally harms human health [3–5]. Some VOCs can even be carcinogenic and genotoxic for humans. Besides humans, VOCs have a harmful effect on the whole environment including flora and fauna [6–8]. It is not surprising that the demand for measuring and monitoring of environmental pollutants has increased in recent years [9]. In industry, VOCs are released primarily from organic solvents, which are frequently used in a wide range of different industrial sectors, like chemical and pharmaceutical plants, painting facilities, *etc.* [10]. Abatement technologies for VOC emissions exist and are sometimes applied. However, the abatement cannot be completely validated, because the crucial point is the lack of accurate, continuous and reliable VOC measurement and monitoring technology. The success of the installed abatement unit is difficult to prove, if the outlet gas of the abatement system cannot be analyzed reliably.

Measuring VOC emissions is challenging. The problem in measuring them is that VOCs can occur in small concentrations (for example in measurements of odorous), but also in very high concentrations. In addition, they show a wide variety in their chemical composition [11–14]. In practice, emission streams are almost always mixtures of several compounds (including moisture and carbon dioxide) whose concentration values are not constant. These facts make the analysis of VOC emissions demanding. Requirements for the measurement system are sensitivity, selectivity and multi component ability. Sometimes the emissions contain corrosive compounds, which make the requirements for the measurement system even tougher. For industrial applications, the system has to be robust and contamination resistant. The presence of water vapor should not influence the measurement, since water is frequently present in industrial measurements. In addition, if the system is used for continuous monitoring or in the scope of process analysis to process control purposes, the system needs to have on-line measurement capability. Until today, there has only been the FT-IR transmission spectroscopy using whitecells, which satisfies most of the requirements mentioned. The transmission technique, however, suffers from certain disadvantages, like the poor stability in a rough and corrosive industrial environment, the non-linear signal response and the high calibration effort. It also suffers from the interference of moisture. In return, photoacoustic spectroscopy has the ability to overcome the limitations mentioned.

By selecting a cantilever enhanced microphone as photoacoustic detector that has been developed in the past few recent years [15–21] photoacoustic spectroscopy, especially the cantilever enhanced one, has several advantages compared to state-of-the-art transmission spectroscopy. One valuable advantage, which can be very useful in industrial emission measurements, is the linearity in signal response. Short optical path lengths of only a few centimeters enable the linear response and opens the door for easy water subtraction, because not only the analyte but also the water absorption behaves linearly [9,18,19,21,22]. The improved photoacoustic detection also provides a linear dynamic range of at least four magnitudes with one point calibration. Together with Science Based Calibration (SBC) [23–25], cantilever enhanced photoacoustic spectroscopy allows low cost calibration and adaptation to different measurement tasks and chemical species. The water subtraction allows accurate process measurements even when water vapor is present, because the water can easily be subtracted and bands, which are overlapping or even lying under the water band can be analyzed [22]. However, the combination of FT-IR and cPAS (cantilever enhanced photoacoustic spectroscopy) was previously realized only for ambient temperatures and up to 50 °C. In some gas measurement applications, especially in industrial emission measurements, the gases to be measured are hot and need to be kept hot in order to avoid condensation. Therefore, the whole measurement system has to be heated. The target of the present approach was to build an FT-IR-cPAS measurement system working at an elevated temperature up to 180 °C and test the sensitivity performance of the system by measuring several different VOCs.

## 2. Experimental Section

### 2.1. FT-IR-cPAS Prototype

The FT-IR-cPAS measurement system consist of three parts, an FT-IR to provide and modulate the light, a photoacoustic cell with an optical cantilever readout (cPAS) to detect the photoacoustic signal and a gas exchange unit to circulate the sample through the measurement system. Bio-Rad’s research grade FTS 6000 was used as FT-IR in the experiments. Since the photoacoustic effect is slow, low frequency modulation, *i.e.*, slow mirror drive, of the IR light is essential in photoacoustic FT-IR spectroscopy. Bio-Rad’s FTS 6000 slowest scanning speed is 2.5 kHz relating to the modulation frequency of the HeNe laser (wavelength of HeNe laser is 632.8 nm, 15,802.8 cm^−1^). To maximize the signal to noise ratio (SNR), the frequency band for the measurement has to be below the resonance frequency of the cantilever. The resonance of the cantilever in the cell is around 4,800 cm^−1^ (∼750 Hz) with a scanning speed of 2.5 kHz. All considerable parameters of the FT-IR are listed in Table 1.

The cantilever enhanced photoacoustic cell (cPA cell) manufactured by Gasera, Finland, was optimized for elevated temperatures. The cell was then integrated into the measurement setup described here. Compared to common photoacoustic detectors, the readout mechanism of the photoacoustic signal is different. Pressure waves, generated in the cell, create a force on the silicon cantilever, the displacement of which is observed optically with an interferometric setup. The position of the cantilever is presented as an analog signal via digital to analog converter and routed to the FT-IR as analog detector interferogram signal. More information about the improved photoacoustic cell, including the detailed principle of operation, quantitative modeling as well as details of the interferometric readout can be found in the following references [15,17–21,26–28]. Table 1 shows the important cell parameters.

The PA cell is optically connected to the FT-IR by an ellipsoidal mirror, which images the focus of the sample compartment to the input aperture of the PAS cell. The light beam leaving the FT-IR has a diameter of 11.94 mm in the focus. The ellipsoidal mirror decreases the beam diameter by 3:1 to 3.98 mm, which is ideal for the PA cell with a diameter of 4 mm. The gas exchange system used was designed, built and tested by VTT. The main effort in designing and building was to find components, which can withstand rather high temperatures (up to 180 °C) and corrosive environment. The corrosion resistance is also important later on in industry, when unknown gases enter the measurement system. In addition, the system should be transportable to be able to carry it to industrial sites. An oven design was chosen to solve the heating problem. All components that needed to be heated were put into the self-built oven. The materials for the parts in contact with the sample gas were chosen to be PTFE or stainless steel grade SS316, sometimes coated with a Silcosteel coating. However, some parts could not be procured in high resistance quality. The function of the gas exchange system is to clean the sample cell by purging it with fresh sample gas, adjusting the pressure of the fresh sample gas inside the cell and after the measurement, purging the cell again with fresh sample gas. For that purpose, the gas exchange unit contains the following parts: 0.5 μm particle filter at the inlet, membrane pump to forward the gas through the system, valves to seal the sample in the photoacoustic cell, a pressure sensor to monitor the sample pressure inside the cell and a control system to monitor the interaction of all components and the temperature inside the oven.

### 2.2. Chemicals—Model VOCs

The need of industry to measure certain VOCs directed the gas selection in this study. The selected model gases and their boiling points are shown in Table 2. All VOCs were measured at the concentrations of 100 ppm and 200 ppm (all the ppm values in this article are given as mol-ppm) diluted in nitrogen. The boiling point is an important value for the measurements because the VOCs are typically liquids in normal conditions and need to be vaporized for the measurement. For the same reason, the compounds can condense easily inside the measurement apparatus if the temperature inside the measurement set-up decreases to a certain level.

### 2.3. Experiments

**Figure 1.**
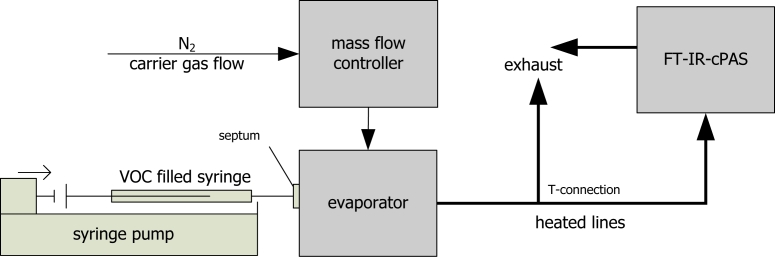
Schematic set up of the VOC vapor generator.

The VOC vapor generator consists of a mass flow controller for adjusting the carrier gas flow, a syringe pump for feeding the organic liquid and a vaporizer to vaporize the liquid. The feed rate of the syringe pump is calculated and adjusted for each VOC and each concentration. The evaporation temperature was chosen to always be 5 °C above the boiling point of the organic liquid. To avoid condensation and to ensure the vaporization, the connection line to the gas exchange system was heated up to 180 °C. For bypassing the sample gas and avoiding overpressures in the system, a T-connection conducted excess gas into exhaust. A scheme of the VOC vapor generator is shown in Figure 1. The sample gas pressure inside the photoacoustic cell was set always set at 1.3 bar.

## 3. Results and Discussion

The first section of this chapter will go into details of the data pre-treatment with the background subtraction as its main issue. It will explain why the background subtraction is important here and how the problem was solved. After that, the second section will expand on the sensitivity of the newly built photoacoustic system. Sensitivity will be analyzed based on the univariate signal to noise ratio (SNR) and the multivariate limit of detection (LoD). The third section will analyze the selectivity and multi component ability by an SBC calibration with five interfering VOCs. In the fourth section, the ability of water subtraction will be tested. Finally, an overall evaluation section will discuss the most important findings. The amplitude of the PA single beam signal is measured in arbitrary units hereafter called PA signal intensity or ‘PAI’ for short.

### 3.1. Data Pre-Treatment

**Figure 2.**
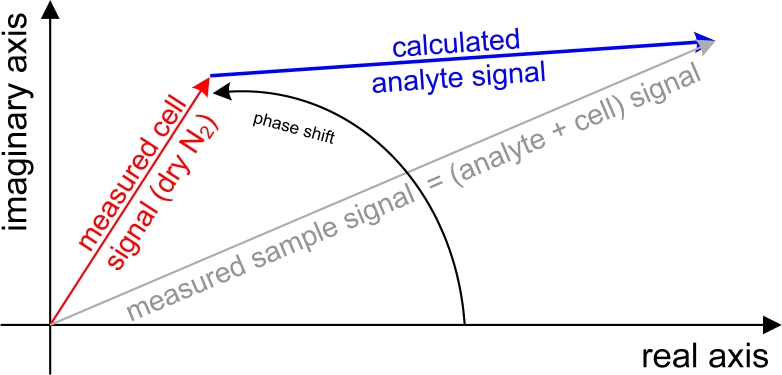
Complex background subtraction strategy at one, arbitrary wavenumber illustrated with vectors in the complex plain. The measured signal with analyte in the cell (grey) contains the signal from both analyte and cell. The measured signal from dry N_2_ (red) only contains the signal from the cell. The desired pure analyte signal (blue) results from the complex background subtraction of the measured cell signal from the measured sample signal.

The output of a Fourier Transformation is a complex vector or in other words a complex spectrum consisting of a real and imaginary part. Calculating the magnitude spectrum via the phase correction [29] is the default setting of the majority of FT-IR software. Three main facts enable the phase correction in conventional transmission spectroscopy: the signal is at a high level at almost all wavelengths, the phase is a ‘slow’ function of the wavenumber and the absorption phenomena taking place in the sample does not affect the signal phase. Else in photoacoustic spectroscopy, the signal is practically zero at wide spectral regions, since only the narrow bands of the sample form the signal. Further, the delay in time between the absorption of the light and the proceeding of the photoacoustic effect, which results in the generation of the pressure wave, creates sample dependent phase changes. For these reasons, the magnitude PA spectrum is typically calculated directly as magnitude value from the real and imaginary parts. Looking from the chemical aspect, the measured PA signal consists of two parts; the signal from the analyte in gas phase and the signal from the cell (background). Since these two phenomena have different time delays or phases, the straightforward subtraction of the magnitude spectrum of the cell lead to incorrect results, especially if the measured photoacoustic signal of the analyte is small. Instead, a complex correction can be used as explained in Figure 2.

The measured interferograms (I) gained from the photoacoustic detector were treated by a complex Fast Fourier Transformation (FFT), giving out the complex signal (S) as real (_r_) and imaginary (_i_) part. To make things easier here, S is the signal at one wavenumber:

(1) $$\text{I}\overset{\text{FFT}}{\to }{\text{S}}_{\text{r},\text{i}}$$

The background signal of the cell, measured with pure, dry nitrogen (Sb_r,i_) (red arrow in Figure 2), is removed by subtracting its real and imaginary parts from the measured sample signal (Ss_r,i_) (grey arrow) resulting in the complex calculated analyte signal Sa_r,i_ (blue arrow):

(2) $${\text{Sa}}_{\text{r},\text{i}}={\text{Ss}}_{\text{r},\text{i}}-{\text{Sb}}_{\text{r},\text{i}}$$

Finally, the magnitude analyte spectrum (Sa_m_) is calculated as power spectrum:

(3) $${\text{Sa}}_{\text{m}}=\sqrt{{\text{Sa}}_{\text{r}}^{2}+{\text{Sa}}_{\text{i}}^{2}}$$

**Figure 3.**
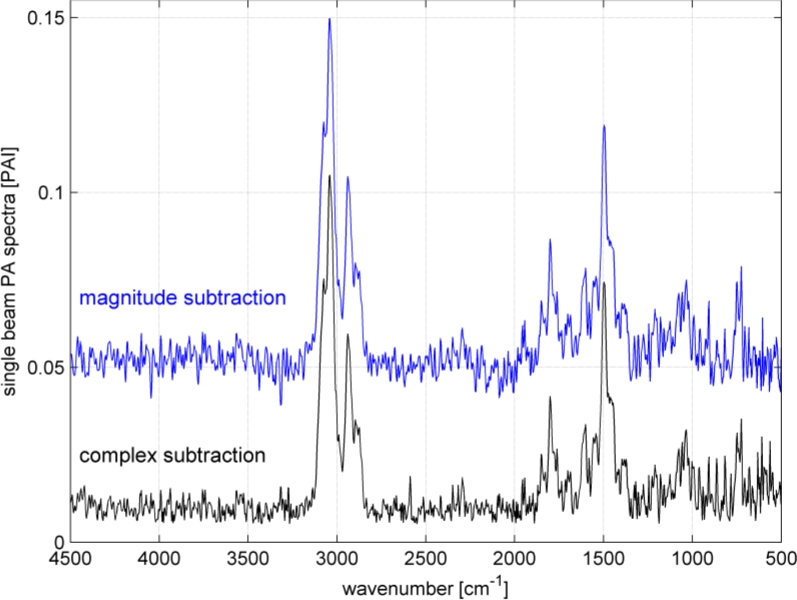
Comparison of the background subtraction performed as complex and magnitude as an example of toluene at 100 ppm. To make this figure well arranged, the result spectrum of the complex subtraction is plotted with an offset of +0.005 PAI and the result spectrum for the magnitude subtraction with +0.05 PAI.

Toluene’s spectrum at 100 ppm was selected to show the differences between the two background subtraction methods. On the one hand, the background was calculated in the complex plain and after that the power spectrum, which will hereafter be called ‘complex subtraction’. On the other hand, the magnitude of the toluene and background spectra were calculated and after that subtracted hereafter called ‘magnitude subtraction’. The visual result of the subtraction is shown in Figure 3.

It can be seen in the figure that the peak heights of the absorption band at 1,500 and 3,000 cm^−1^ are identical independent of the subtraction method used. However, the baseline of the spectrum resulting from the complex subtraction is smoother and the amplitude of the noise seems to be smaller. This visual observation can be proven by calculating the coefficient of variation (CV) of the spectral regions where no absorption occurs. It turns out that the CV is smaller by a factor of 3.5 for the complex subtraction than for the magnitude in the spectral range between 2,200 and 2,800 cm^−1^. Still, for both subtraction methods the background in the region from 500 to 1,400 cm^−1^ looks somehow higher than the background in the region between 2,200 and 2,800 cm^−1^. This is due to two weak pronounced toluene absorption bands, the C-H in plane bending (1,000 to 1,100 cm^−1^) and the C-H out of plane bending (720 to 820 cm^−1^). Those two absorption bands are slightly higher than the surrounding noise and hence impute a higher noise level.

In photoacoustic spectroscopy, when no phase correction can be performed, the background should be subtracted in the complex plain. In this way, higher precision is achieved resulting in smaller noise residuals in the spectrum and a higher signal to noise ratio, compared to the magnitude background correction. Still, since the power spectrum is used at the final stage, the method suffers from the fact that the noise in absolute values cannot become negative numbers, which shifts the spectrum to slightly higher values on the ordinate. The slight offset shift can be corrected with an offset correction.

### 3.2. Single Component Analysis

The signal to noise ratio (SNR) is calculated by dividing the univariate signal S by the noise N. The standard deviation of each VOC spectrum was calculated in the region from 2,400 to 2,800 cm^−1^. Because the amount of data points was too small to make a precise noise estimation (51 optical resolved points), all the calculated standard deviation values were averaged. The signal and the noise are given in Table 3. N is the RMS noise with the magnitude of one standard deviation (1σ). The equivalent measurement time for each VOC of 900 averaged scans was 25.5 min at a resolution of 8 cm^−1^.

The calculated SNR values for the 18 VOCs varies a lot, from 19 (the lowest) for o-xylene to 329 (the highest) for butyl acetate. SNR is a meaningful parameter to describe the relation of the signal to the noise. What does for o-xylene mean: The univariate signal of 100 ppm o-xylene at 2,940 cm^−1^ is 19 times larger than the estimated noise between 2,400 and 2,800 cm^−1^.

Calculating univariate characterization parameters such as the SNR presented here downgrades the performance of the FT-IR-cPAS. This is due to FT-IR-cPAS being a multivariate measurement instrument which measures the photoacoustic signal at several and not just at a single wavenumber. An analyte band spreading over several wavenumbers, is underestimated in the univariate (SNR) case, because the gained information about the photoacoustic signal at all the other wavenumbers (the rest of the photoacoustic spectrum) is neglected. The multivariate limit of sensitivity should be used to calculate the limit of detection (LoD) in spectroscopy. Equation (4) is a part of the recently presented science based method or science based calibration (SBC). More information about the SBC and its mathematical derivation can be found in [23–25]:

(4) $$\text{BEC}=\sqrt{\frac{1}{g^{T}\cdot \sum^{-}\cdot g}}$$

where BEC is the background noise equivalent concentration [ppm], ∑^−^ the covariance matrix of the noise [PAI^2^], g the response spectrum of the analyte as column vector [PAI·ppm^−1^] and g^T^ the response spectrum of the analyte as row vector [PAI·ppm^−1^]. The International Union for Pure and Applied Chemistry (IUPAC) defined the LoD as follows: “The limit of detection is derived from the smallest measure that can be detected with reasonable certainty for a given analytical procedure” [30]. Whereby, 3 standard deviations (3σ) are recommended for calculating the LoD [31]. The case when the measured signal has the same magnitude as the noise (1σ) is called background noise equivalent concentration (BEC).

The diagonal of the ∑ matrix was filled with the smoothed standard deviation of 3 measured dry nitrogen spectra. ∑ was computed from the instrument noise; no other interference or noise source than the sampling noise was taken into account. Hence, the LoD values presented here will be discussed as best possible ones for the FT-IR-cPAS. The noise was determined with 300 scans which corresponds to a measurement time of 8.5 min at a resolution of 8 cm^−1^. For both the noise and the analyte signal, the full spectral area from 500 to 4,500 cm^−1^ was used. Table 4 shows the LoD (3σ) for each VOC. One more interesting parameter is the comparison between uni- and multivariate LoD, or in other words how much the multivariate LoD performs better. First, the univariate LoD is calculated as:

(5) $$\text{univariate}\;\text{LoD}=\frac{\text{gas}\;\text{concentration}}{\text{SNR}}=\frac{10\;\text{ppm}}{\text{SNR}[]}$$

The LoD ratio, which can be found in Table 4, relates the univariate LoD with the multivariate BEC (each 1σ) as:

(6) $${\text{LoD}}_{\text{ratio}}=\frac{{\text{LoD}}_{\text{univariate}}[\text{ppm}]}{{\text{BEC}}_{\text{multivarite}}[\text{ppm}]}.$$

The LoD data in Table 4 is pessimistic because of a numerical particularity of FT-instruments. Before FT transformation, the interferogram is usually appended with zeros to the largest power-of-2 number (…512, 1,024, 2,048…). This enables efficient computation using the Fast Fourier Transformation (FFT) algorithm but also interpolates the resulting spectral data points. In other words, neighboring spectral points are not independent from each other, since even the high frequency electronic noise (affecting the interferogram) has been interpolated in the spectra. This could be described by putting non-zero elements on the side diagonals in the noise matrix ∑. To avoid this time-consuming step, the LoD is calculated with empty side diagonals (as explained above). Then, the correction factor f in Equation (7) has to be taken into account to become accurate again:

(7) $$\text{factor}\;\text{f}=\sqrt{\frac{\text{numerical}\;\text{FFT}\;\text{points}}{\text{optical}\;\text{resolved}\;\text{points}}}=\sqrt{\frac{2048}{987}}=\sqrt{2.07}$$

**Figure 4.**
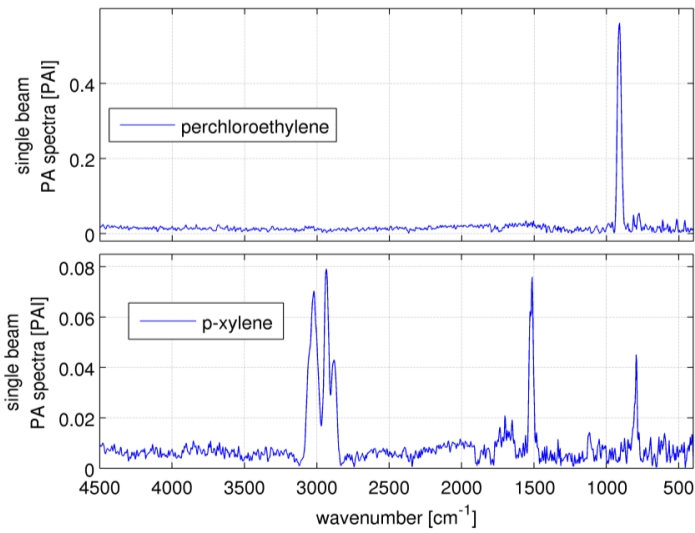
Two extreme cases for multivariate data analysis: spectra of perchloroethylene (PCE) and *p*-xylene. Perchloroethylene shows one fine absorption band, which does not gain that much from multivariate data analysis. *Vice versa*, *p*-xylene gains from multivariate analysis, because its spectrum has tiny but several absorption bands, from which one is relatively broad.

Hence, the expected LoD values are better by factor ≈ $\sqrt{2}$ than the ones stated here. Multivariate analysis improves the sensitivity relative to univariate analysis because, graphically speaking, the sensitivity of many wavelengths is “added up”. The best possible sensitivity for a certain wavenumber range is given by Equation 4 and in practice achieved by so-called “matched filter” calibration [23–25]. Table 4 shows the improvements, which are between 2.2 for perchloroethylene and 9.2 for *o*-xylene. The multivariate method gains from more and broader signal bands. Figure 4 shows the spectra of perchloroethylene and *p*-xylene. Perchloroethylene’s spectrum shows only one fine absorption band, which is covered by 16 data points. Making a generalization, the fine band almost represents the univariate case itself. The factor of improvement is low. An opposite extreme is *p*-xylene, where the spectral features are relatively broad but tiny and slightly larger than the noise level. This case gains from the relative broad band around 3,000 cm^−1^ covered by 69 data points.

The LoD numbers are adequate according to the emission limits stated by Directive 2010/75/EU. Directive 2010/75/EU appoints the emission limit of 20 mg·Nm^−3^ for VOCs with the hazard statement H341 or H351 (earlier R-label R40 and R68) and 2 mg·Nm^−3^ stated with H340, H350, H350i, H360D or H360F (earlier R45, R46, R49, R60 and R61) (Nm^3^ stands for norm cubic meter and refers to a temperature of 273.15 K and a pressure of 101.3 kPa) [32]. Three of the model VOCs fall under the regulation of Directive 2010/75/EU. Table 5 shows the VOCs, their H-statement, emission limit and experimentally gained LoD.

The presented detection limits are only true if no other spectral interference or noise component is present. If other components such as other VOCs are present and interfering (overlapping the spectra) the detection limit will increase. The next section will evaluate the interferences of analytes in a multi component mixture.

### 3.3. Multi Component Analysis

**Figure 5.**
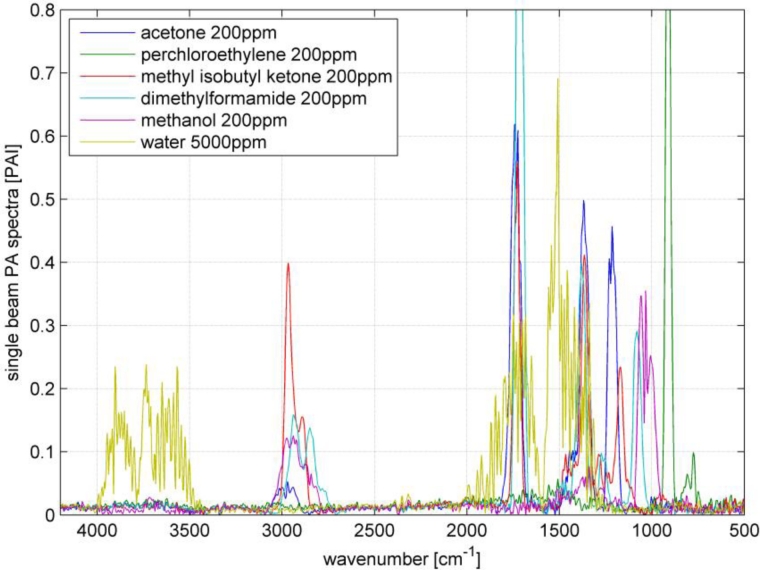
Selectivity experiment: Spectra of the five VOCs and water.

**Figure 6.**
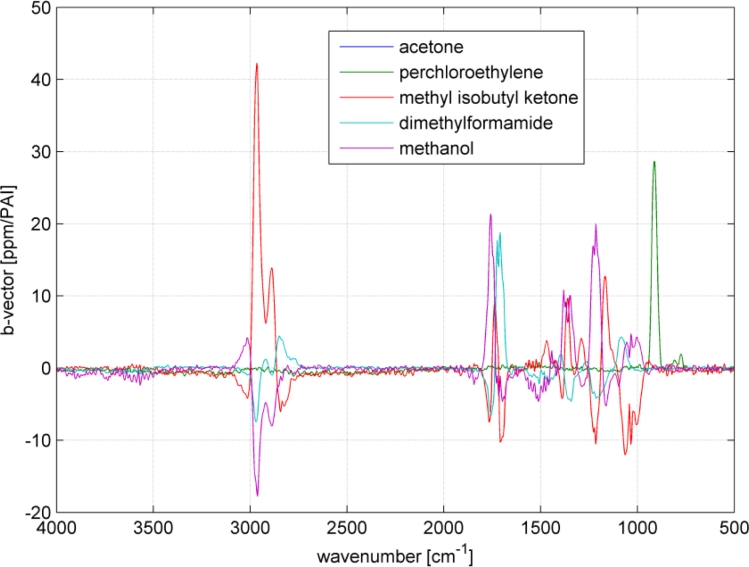
Selectivity experiment: b-vectors of the five calibrations.

Multi component ability and selectivity (*i.e.*, the interferences between the analytes) will be shown with an SBC calibration. Five VOCs were selected to set up a quantitative multi component calibration. The VOCs were acetone, perchloroethylene, methyl isobutyl ketone, dimethylformamide and methanol. In addition, water was added as an interferent, since it is frequently present in industrial measurements. Figure 5 shows the spectra of the five selected VOCs and water. The calibration was set up with VOC spectra of 200 ppm and water of 5,000 ppm. For each VOC, one SBC calibration was set up including the interference noise of the four other VOCs and water. The standard deviation of the interfering VOCs (how much the concentration of the interferent can change in the subsequent measurements) was set to 500 ppm and water 1,000 ppm. Further, the noise matrix contained the hardware noise floor and offset noise. The calculated b-vectors alias regression vectors are shown in Figure 6.

**Figure 7.**
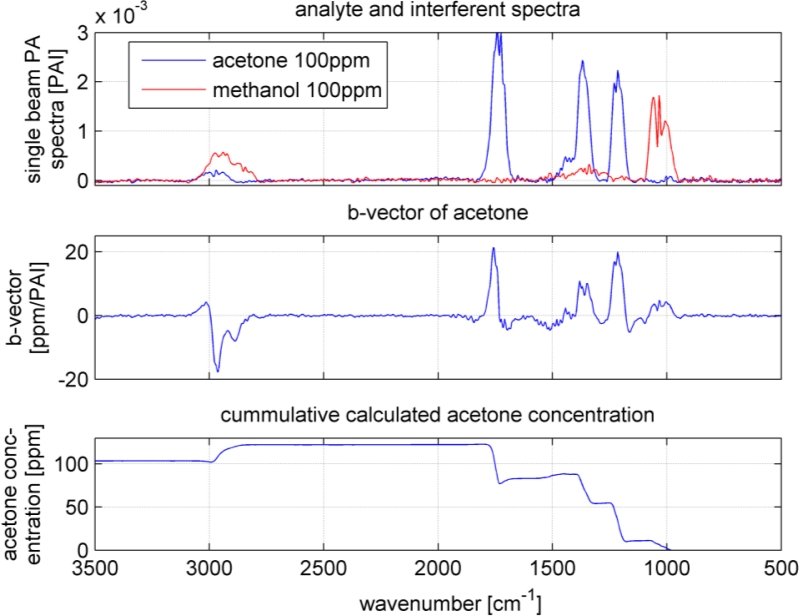
Sample spectrum, b-vector and result calculation: A schematic demonstration. The upper graph shows the sample spectrum (analyte and interferent spectra plotted separate), in the middle the b-vector for the analyte acetone and the lower graph the resulting cumulative sum of the vector multiplication of b-vector and sample spectrum (accumulation starts from 500 cm^−1^).

As it can be seen in Figure 5, the spectra overlap heavily. However, the b-vectors contain negative elements, which will cancel out the interferences. Figure 7 shows an example how a b-vector and its multiplication ‘work’. In this example the concentration of the analyte acetone will be calculated using the b-vector of acetone that includes the interferent information of all four interfering VOCs. To keep the overview and not make this example to complicated only methanol was chosen as interferent. The sample gas contains 100 ppm of acetone and 100 ppm of methanol (spectra in upper graph in Figure 7). The measured sample gas spectrum will be multiplied with acetone’s b-vector to achieve the sample’s acetone concentration. Dependent on the shape and the amplitude of the b-vector and the spectrum, the concentration accumulates at each wavenumber. The lower graph in Figure 7 shows the accumulated multiplication curve starting from 500 cm^−1^ and ending at 3,500 cm^−1^. The concentration increases with the analyte bands at 1,200, 1,350 and 1,750 cm^−1^. However, the methanol band at 1,050 cm^−1^ lifted the concentration too high, which is compensated by the negative b-vector elements at 2,900 cm^−1^ resulting in an acetone concentration of 102 ppm.

A numerical expression of the selectivity is the cross selectivity, which is calculated between the five VOCs. The 100 ppm spectra of the VOCs are divided by 100 and multiplied by the b-vector of each VOC. Table 6 shows the calculated cross selectivities. A cross selectivity of 0.10 (10%) means, if the interferent changes e.g., by 100 ppm, the analyte concentration will change by 10 ppm.

Most of the pairs show cross selectivities below 0.01 (1%). Four pairs have 2% and three exceptions which are >2%. The average cross selectivity is <2%. The calibration is pretty immune against water, since the water cross selectivities are below 0.2%. Due to the additional interference noise, the detection limits have changed. Table 7 shows the detection limits for the multi component analysis and compare it with the single component measurements. The detection limits went up for all VOCs due to the overlapping of the spectra. Acetone shows the highest increase of factor, 2.2. The detection limit of the four other VOCs have not increased by more than a factor of 2.

### 3.4. Water Subtraction

**Figure 8.**
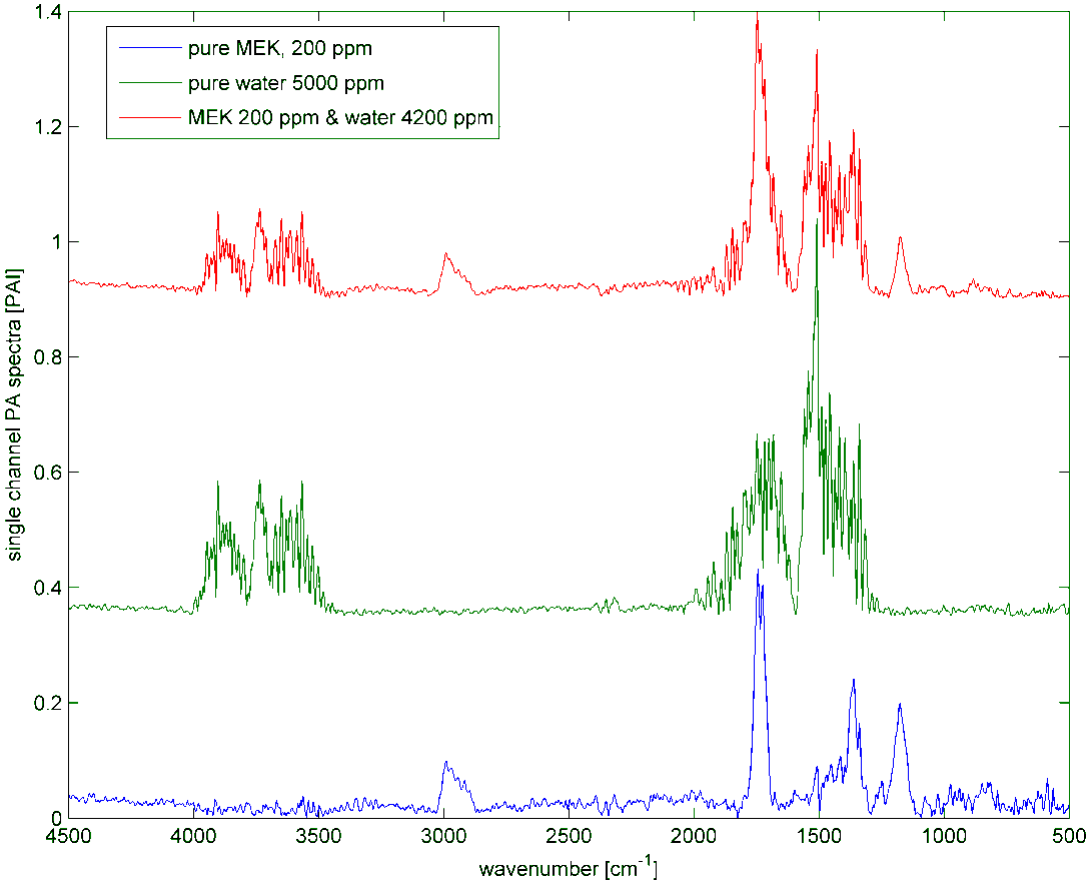
Demonstration of water overlapping with the analyte: If the pure MEK sample (blue) contains water (green), the measured spectra will be the sum of both (red).

**Figure 9.**
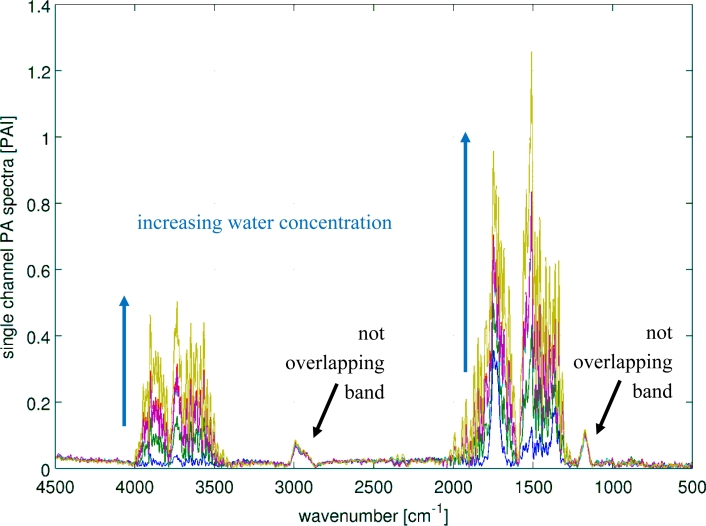
Water subtraction experiment: MEK concentration was always 200 ppm while the water concentration were 1,400, 4,200, 12k, 35k and 100k ppm.

**Figure 10.**
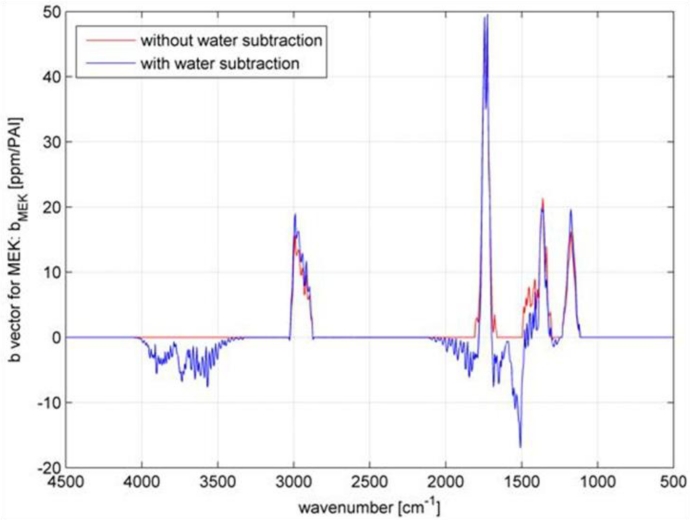
Water subtraction experiment: b-vectors. The blue b-vector was calculated with water as an interferent. It shows negative elements, which will cancel out the interference of water. The red b-vector, without the information of water interference, does not show negative elements.

Humid samples are a major challenge in the analysis of IR spectra, when the spectrum of water overlaps the spectrum of the analyte as seen in Figure 8. Still, to be able to use the overlapping region for data analysis, in particular quantitative data analysis, the water has to be subtracted. In this experiment, the concentration of methyl ethyl ketone (MEK) was always 200 ppm, while water was added to the samples in concentrations spreading from 1,400 ppm to 100k ppm. The measured spectra are shown in Figure 9. The subtraction of water was done with a SBC calibration, where MEK was the analyte of interest. One water spectra (10k ppm) was added as an interferent in the calibration, so that the b-vector will cancel out the water features and predict the true MEK concentration. A second calibration was set up without adding water as an interferent. Both b-vectors are shown in Figure 10.

The results of the water subtraction experiment are shown in Table 8. If the interference of water is not cancelled out by the calibration, the calculated MEK concentrations increase with increasing water concentrations. If the information of the water interference is added to the calibration, it will calculate the true MEK concentration with a coefficient of variation (CV) of 3%.

The variation of the calculated MEK concentration is not induced by the calibration method. The variation seen here can be explained by the experimental deviation of the true MEK concentration, since the CV of the area of the non-overlapping band (2,850–3,050 cm^−1^) is 4.5%. The variation in the MEK concentration can be explained by the gas feeding system, which may have several points of uncertainty. One possibility can be the time instability of the syringe feed, which would cause direct changes in the true analyte concentration.

### 3.5. Overall Evaluation

Reflecting back to the introduction, the listed needs for an industrial emission measurement system are: selectivity, sensitivity, multi component ability, corrosion resistance, high measurement temperature, low influence of water vapor, online capability and robustness. The temperature was successfully increased to 180 °C, which is high enough for emission measurements. Corrosion resistance was realized on a basic level, since all components were SS316. Better corrosion resistance (PTFE, Silcosteel coating) was achieved for some parts, but a few (e.g., the valves) were SS316.

For single component measurements, the detection limits were in compliance with the statutory emission limits. For the five component mixture with water, the detection limits only increased by a maximum factor of 2.2. Still, the gained sensitivity couldn’t reach the state of the art (too long measurement time), which is due to the non optimal alignment and coupling of the cell to the FT-IR. In these experiments, a high resolution FT-IR was used. By having a high resolution spectrometer, the aperture is limited to a certain size, which is, on the other hand, the bottleneck for sensitivity. Bio-Rad’s FT-IR has a maximal aperture size for 4 cm^−1^ of resolution (11.94 mm), although the spectra were measured with 8 cm^−1^ resolution, where light power was lost. In future, the sensitivity can be increased by selecting a low resolution FT-IR with a much higher light throughput.

The low cross selectivities of the five component calibration and the successful water subtraction showed that the resolution of 8 cm^−1^ is still good enough to offer selectivity. By increasing the resolution (e.g., to 4 cm^−1^ or even better), the cross selectivities might improve, but the SNR will drop down for the same measurement time. An application specific tradeoff between selectivity and sensitivity has to be found. For the case presented here, the resolution better than 8 cm^−1^ was not needed.

The presence of water influenced the calibration less than 0.2%. The water subtraction was studied in more detail and the subtraction turned out to be accurate (within a CV of 3%) with only one water ‘library’ spectrum. This is a big benefit for measurement applications where water is present, since no complex water libraries are needed and the subtraction itself is easier due to the linear behavior (scaling of the 10k ppm subtraction spectrum fit the 1,400 ppm as well as the 100k ppm).

In principle, the device is ready for process analysis, although the measurement time needs to be decreased in the upcoming investigations (optimization of the FT-IR coupling). One drawback is the restriction of the non continuous flow, *i.e.*, the gas flow needs to be stopped and the valves closed for measurement. This is a disadvantage for continues monitoring and for certain gases due to possibly occurring adsorption phenomena especially when the cell is not heated. The last point is the robustness for industrial use. Since this is difficult to evaluate in a laboratory, further studies are planned to test the system under real industrial conditions.

## 4. Conclusions

Photoacoustic FT-IR spectroscopy was successfully brought to high temperatures up to 180 °C. The performance of the novel heated FT-IR-cPAS system was studied by laboratory VOC measurements. It turned out that a complex background correction has to be performed to correct the phase shift of the photoacoustic signal after the FFT. Sensitivity was explored as univariate SNR (1σ) and multivariate LoD (3σ). The multivariate analysis using SBC was up to 9.2 times better compared to the univariate analysis (both 1σ). SNR (1σ) numbers for the 18 measured VOCs were varying between 19 (the lowest) for *o*-xylene and 329 (the highest) for butyl acetate at a measurement time of 25.5 min. In the same way, the multivariate LoD (3σ) varied between 2.60 ppm (worst) for dichloromethane to 0.33 ppm (best) for butyl acetate within 8.5 min. The LoDs of the VOC were in compliance with the statutory emission limits stated by Directive 2010/75/EU for single compound measurement. Selectivity and multi component ability were shown by an SBC calibration with 5 VOCs and water. On visual inspection, the six spectra overlapped heavily. Still, the cross selectivity (the numerical expression of the selectivity) could be kept below 2% for most of the interference pairs. The resulting detection limits increased by a maximum factor of 2.2. The successful subtraction of water could be shown by another SBC calibration which calculated the true analyte concentration with a variation coefficient of 3%, although the variation in the water concentration covered almost three magnitudes (1,400 to 100k ppm) and the used subtraction water spectrum had the concentration of 10k ppm. Even though the FT-IR-cPAS technology shows some weaknesses (e.g., the sample gas stream needs to be stopped for the measurement) it provides features which are superior compared to transmission spectroscopy as the water subtraction ability or the easiness of calibration. Therefore it is worth, developing it further to reach an industrial ready technology.

## Figures and Tables

**Table 1.** Instrument parameters of FT-IR and cPAS cell.

**Parameter**	**Value**	**Parameter**	**Value**
FT-IR interferometer			

Manufacturer	Bio-Rad	model	FTS 6000
Resolution	8 cm^−1^	mirror velocity	2.5 kHz
Spectral Range	400–8,000 cm^−1^	beam splitter	KBr
Aperture	11.94 mm	focal spot size	11.94 mm
Co-Added Scans	300		

Photoacoustic Sample Gas Cell			

Manufacturer	Gasera	model	PA101h
Material	stainless steel, inside gold	gas volume	about 8 mL
Diameter	4.0 mm	length	100 mm
Internal Geometry	cylindrical	optical path length	200 mm
Window Diameter	13 mm	temperature range	15–200 °C
Window Material	BaF_2_	sample pressure	0–2 bar
Resonant Mode	non-resonant		

Cantilever			

Material	silicon, gold coated	thickness	10 μm
Length	5 mm	resonance frequency	750 Hz
Width	1.2 mm	gap between frame and cantilever	<5 μm

**Table 2.** Model VOCs used in the experiments.

**VOC**	**Boiling point**	**VOC**	**Boiling point**
acetone	56	methoxypropanol acetate	146
*n*-butanol	117	methyl acetate	60
butyl acetate	126	methyl ethyl ketone	80
dichloromethane	40	methyl isobutyl ketone	118
dimethylformamide	153	perchloroethylene	121
ethanol	78	toluene	111
isobutanol	108	*o*-xylene	144
isopropanol	83	*m*-xylene	139
methanol	65	*p*-xylene	138

**Table 3.** Signal to noise ratio (SNR) and its calculation parameters for each VOC: wavelength where the signal was taken and corresponding signal height. N is the RMS noise of the region 2,400–2,800 cm^−1^ with the magnitude of one standard deviation (1σ). N is for all VOCs 3.53e–3, since the standard deviation was averaged over all VOCs. The concentration of each VOC was 100 ppm.

**VOC**	**Signal at wave-number [cm^−1^]**	**Signal [PAI]**	**SNR**
acetone	1,744	4.02e–01	114
ethanol	1,053	1.08e–01	31
isobutanol	1,042	2.21e–01	63
isopropanol	2,978	1.73e–01	49
methanol	1,057	1.59e–01	45
*n*-butanol	2,943	2.07e–01	59
perchloroethylene	910	5.61e–01	159
methoxypropanol acetate	1,242	1.12e+00	316
methyl acetate	1,246	8.18e–01	232
methyl ethyl ketone	1,744	2.16e–01	61
methyl isobutyl ketone	1,724	2.68e–01	76
*o*-xylene	2,940	6.71e–02	19
*m*-xylene	2,940	6.71e–02	19
*p*-xylene	1,508	7.33e–02	21
dimethylformamide	1,724	7.27e–01	206
dichloromethane	1,277	1.21e–01	34
butyl acetate	1,234	1.16e+00	329
toluene	3,040	1.00e–01	28

**Table 4.** LoD for each measured VOC as 3σ. For both the noise and the analyte signal, the full spectral area from 500 to 4,500 cm^−1^ was used. The concentration of each VOC was 100 ppm. The LoD ratio relates the uni- with the multivariate LoD and is an indicator of how much better the multivariate LoD performs.

**VOC**	**LoD (3σ) [ppm]**	**LoD ratio: (uni/multi)variate [ ]**
acetone	0.55	4.9
ethanol	1.70	5.9
isobutanol	0.83	5.7
isopropanol	1.00	6.2
methanol	1.50	4.4
*n*-butanol	0.81	6.3
perchloroethylene	0.85	2.2
methoxypropanol acetate	0.33	2.9
methyl acetate	0.36	3.6
methyl ethyl ketone	1.10	4.3
methyl isobutyl ketone	0.83	4.7
*o*-xylene	1.70	9.2
*m*-xylene	1.80	8.8
*p*-xylene	1.90	7.8
dimethylformamide	0.56	2.9
dichloromethane	2.60	3.4
butyl acetate	0.33	3.0
toluene	1.70	6.1

**Table 5.** Emission limits according Directive 2010/75/EU and the experimentally achieved LoD with a measurement time of 8.5 min.

**VOC**	**H-statement**	**Emission limit concentration [mg**·**Nm^−3^]**	**Emission limit [ppm]**	**LoD (3σ) [ppm]**
dichloromethane	H351	20	5.5	2.60
dimethylformamide	H360D	2.0	0.6	0.56
perchloroethylene	H351	20	2.8	0.85

**Table 6.** Cross selectivity’s of the five VOCs and water in [ppm·ppm^−1^]. For example, when measuring acetone and perchloroethylene’s concentration increases by 100 ppm, the measured acetone value will decrease by 1 ppm.

	
	**Interferent**						

**analyte ↓**	**acetone**	**perchloroethylene**	**methyl isobutyl ketone**	**dimethylformamide**	**methanol**	**water**	**sum**

acetone	1.00	−0.010	0.008	0.002	0.020	<0.001	0.037
perchloroethylene	−0.012	1.00	−0.007	<0.001	0.008	<0.001	0.029
methyl isobutyl ketone	−0.004	−0.020	1.00	−0.067	−0.011	0.002	0.098
dimethylformamide	0.005	<0.001	−0.004	1.00	−0.020	−0.002	0.036
methanol	−0.013	0.002	−0.034	−0.086	1.00	0.004	0.130

**Table 7.** Comparison of the detection limits: single *versus* and multi component. The single component detection limits (Table 4) were calculated without interference noise. The multi component detection limits including the interference of 4 other VOCs and water.

**VOC**	**LoD (3σ) [ppm]**	

	**Single**	**Multi**
acetone	0.55	1.20
methanol	1.50	1.85
perchloroethylene	0.85	1.00
methyl isobutyl ketone	0.83	1.41
dimethylformamide	0.50	0.71

**Table 8.** Water subtraction experiment: results of the analysis using the water subtracted and not water subtracted calibration.

**MEK concentration [ppm]**	**Water concentration [ppm]**	**Calculated MEK concentration [ppm]**	

		**without subtraction**	**with subtraction**
200	1,400	238	202
200	4,200	336	196
200	12k	481	198
200	35k	453	187
200	100k	650	200
